# The modulation of leguminous plant ethylene levels by symbiotic rhizobia played a role in the evolution of the nodulation process

**DOI:** 10.1016/j.heliyon.2018.e01068

**Published:** 2018-12-20

**Authors:** Francisco X. Nascimento, Maria J. Tavares, Márcio J. Rossi, Bernard R. Glick

**Affiliations:** a Departamento de Microbiologia, Universidade Federal de Santa Catarina, Florianópolis, SC, 88040-900, Brazil; b Department of Biology, University of Waterloo, Waterloo, ON N2L 3G1, Canada

**Keywords:** Biotechnology, Ecology, Microbiology, Plant biology, Evolution

## Abstract

Ethylene plays an important role in regulating the rhizobial nodulation process. Consequently, numerous strains of rhizobia possess the ability to decrease plant ethylene levels by the expression of the enzyme 1-aminocyclopropane-1-carboxylate (ACC) deaminase or via the production of rhizobitoxine, thus, leading to an increased ability to nodulate leguminous plants. Nevertheless, not much is understood about the prevalence of these ethylene modulation genes in different rhizobial groups nor their role in the evolution of the symbiotic process.

In this work, we analyze the prevalence and evolution of the enzymes ACC deaminase (AcdS) and dihydrorhizobitoxine desaturase (RtxC) in 395 NodC^+^ genomes from different rhizobial strains isolated from a wide range of locations and plant hosts, and discuss their importance in the evolution of the symbiotic process.

The obtained results show that AcdS and RtxC are differentially prevalent in rhizobial groups, indicating the existence of several selection mechanisms governed by the rhizobial strain itself and its evolutionary origin, the environment, and, importantly, the leguminous plant host (co-evolution). Moreover, it was found that the prevalence of AcdS and RtxC is increased in *Bradyrhizobium* and *Paraburkholderia*, and lower in other groups. Data obtained from phylogenetic, evolutionary as well as gene localization analysis support the previous hypotheses regarding the ancient origin of the nodulation abilities in *Bradyrhizobium* and *Paraburkholderia,* and brings a new perspective for the importance of ethylene modulation genes in the development of the symbiotic process. The acquisition of AcdS by horizontal gene transfer and a positive selection in other rhizobial groups indicates that this enzyme plays an important role in the nodulation process of many rhizobia. On the other hand, RtxC is negatively selected in most symbioses.

Understanding the evolution of ethylene modulation genes in rhizobia may be the key to the development of new strategies aiming for an increased nodulation and nitrogen fixation process.

## 1 Introduction

In the leguminous plant-rhizobia symbiosis, rhizobia produce lipochitooligosaccharides, termed Nodulation (Nod) factors (NFs), encoded by *nod* genes that induce the plant's symbiotic response. On the other hand, leguminous plants produce various flavonoids, that induce *nod* gene expression in rhizobia, and perceive and respond to different rhizobial NFs, therefore, controlling the success of bacterial internalization and nodule formation. Once inside the root nodule, efficient rhizobia fix atmospheric nitrogen and provide it to the plant host. Hence, the rhizobial nodulation process is of extreme importance for agricultural and biotechnological processes (Gage, 2004).

Ethylene is a central regulator of the nodulation process (Berrabah et al., 2018; Guinel, 2015; Larrainzar et al., 2015). In all higher plants, ethylene is produced from 1-aminocyclopropane-1-carboxylate (ACC) by the action of the enzymes ACC synthase and ACC oxidase. Downstream, the ethylene signaling pathway is comprised of several elements, which, ultimately, lead to the activation of ethylene-induced transcription factors that modulate the expression of several genes, including those involved in the nodulation process and plant defense responses (Guinel, 2015; Nascimento et al., 2018).

Bacteria have evolved intricate mechanisms to modulate plant ethylene levels, either through the production of the enzyme ACC deaminase (Glick et al., 1998) or the vinylglycine compound rhizobitoxine (RTX) (Sugawara et al., 2006). The enzyme ACC deaminase, encoded by the *acdS* gene, degrades ACC, the ethylene precursor, into ammonia and alpha-ketobutyrate (Glick et al., 1998). Rhizobia expressing ACC deaminase reduce the negative effects of ethylene in the nodulation process (reviewed in Nascimento et al., 2016). Rhizobitoxine (RTX) is a secreted enol-ether amino acid that acts as an inhibitor of the plant ACC synthase (Yasuta et al., 1999), leading to a reduction in ACC production and, consequently, decreased plant ethylene levels (Yuhashi et al., 2000). The dihydrorhizobitoxine desaturase enzyme, encoded by the *rtxC* gene, is involved in the ultimate and vital step that leads to the formation of RTX (Okazaki et al., 2004). *Bradyrhizobium* strains impaired in RTX production form fewer nodules in their host plants (Duodu et al., 1999) and are less competitive than their wild-type counterparts (Okazaki et al., 2004; Ratcliff and Denison, 2009; Yuhashi et al., 2000).

The symbiotic compatibility and plant infection control mechanisms have led to a selection pressure on the bacterial symbionts over millions of years of evolution. In this sense, the diversity of *nod* genes (including the *nodC* gene that encodes a N-acetylglucosaminyltransferase involved in NF production) and NFs produced by rhizobia is an indication of rhizobia adjusting to plants (Martinez-Romero, 2009). Moreover, the specificity of the interaction between *nod*-containing rhizobia and their cognate leguminous plants allows for the study of leguminous host effect in rhizobial evolution.

Since ethylene plays a central role in plant defense responses and the nodulation process, it is hypothesized that ethylene modulation mechanisms such as ACC deaminase and rhizobitoxine production may be selected (positively or negatively) in rhizobia (*sensu lato*). Nevertheless, not much is understood about the prevalence of these mechanisms in rhizobia, the factors mediating their evolution and their possible selection by co-evolution with specific plant hosts. Hence, in this work, through a bioinformatics approach, the prevalence of ethylene modulation genes was studied in the genomes of 395 NodC-containing rhizobia isolated from a wide range of plant hosts and countries. In addition, the evolution of AcdS and RtxC was analyzed based on phylogenetic and evolutionary distances comparisons with NodC and the housekeeping DNA recombinase A (RecA) proteins and gene localization analysis. Based on this data we present and discuss the evidence for the selection of ethylene modulation genes in rhizobia and its role in the development/evolution of the symbiotic process.

## 2 Results

### 2.1 Phylogenetic and evolutionary analysis of NodC in completely sequenced bacterial genomes

A total of 395 NodC-containing genomes belonging to several bacterial groups and isolated from several leguminous plant hosts in several countries were identified (Table S1). From Alphaproteobacteria the following strains were found: *Allorhizobium* (n = 1)*, Azorhizobium* (n = 2)*, Bradyrhizobium* (n = 71), *Mesorhizobium* (n = 96), *Microvirga* (n = 3), *Methylobacterium* (n = 3)*, Rhizobium* (n = 104), *Neorhizobium* (n = 11)*, Pararhizobium* (n = 1)*, Sinorhizobium/Ensifer* (n = 71); From Betaproteobacteria: *Paraburkholderia* (n = 27), *Cupriavidus* (n = 5). No NodC-containing genomes belonging to other known nodulating bacterial species (e.g. *Bosea*, *Devosia, Phyllobacterium, Ochrobactrum, Aminobacter* or *Pseudomonas*) were found in the NCBI database.

**Fig. 1:**
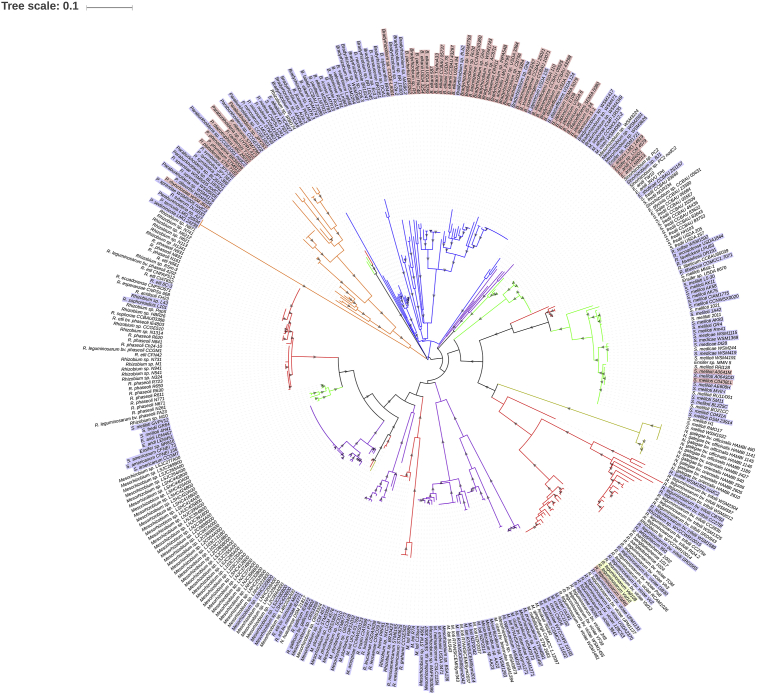
Phylogram based on NodC sequences from rhizobia. The evolutionary history was inferred by using the Maximum Likelihood method based on the JTT matrix-based model (Jones et al., 1992). The tree with the highest log likelihood (-20346.9200) is shown. Initial tree(s) for the heuristic search were obtained by applying the Neighbor-Joining method to a matrix of pairwise distances estimated using a JTT model. A discrete Gamma distribution was used to model evolutionary rate differences among sites ((+G, parameter = 0.9717)). The rate variation model allowed for some sites to be evolutionarily invariable ([+I], 24.4911% sites). The tree is drawn to scale, with branch lengths measured in the number of substitutions per site. The presence of grey triangles in the branches indicates a bootstrap support from 50 to 100%. The blue labels indicate the sole presence of AcdS in the strain. Red labels indicate the presence of both AcdS and RtxC in the strain. Yellow labels indicate the sole presence of RtxC in the strain. *Bradyrhizobium*- blue branches; *Paraburkholderia*- orange branches; *Mesorhizobium*- purple branches; *Rhizobium*- red branches; *Sinorhizobium/Ensifer*- green branches; *Neorhizobium*- light brown branches.

**Fig. 2:**
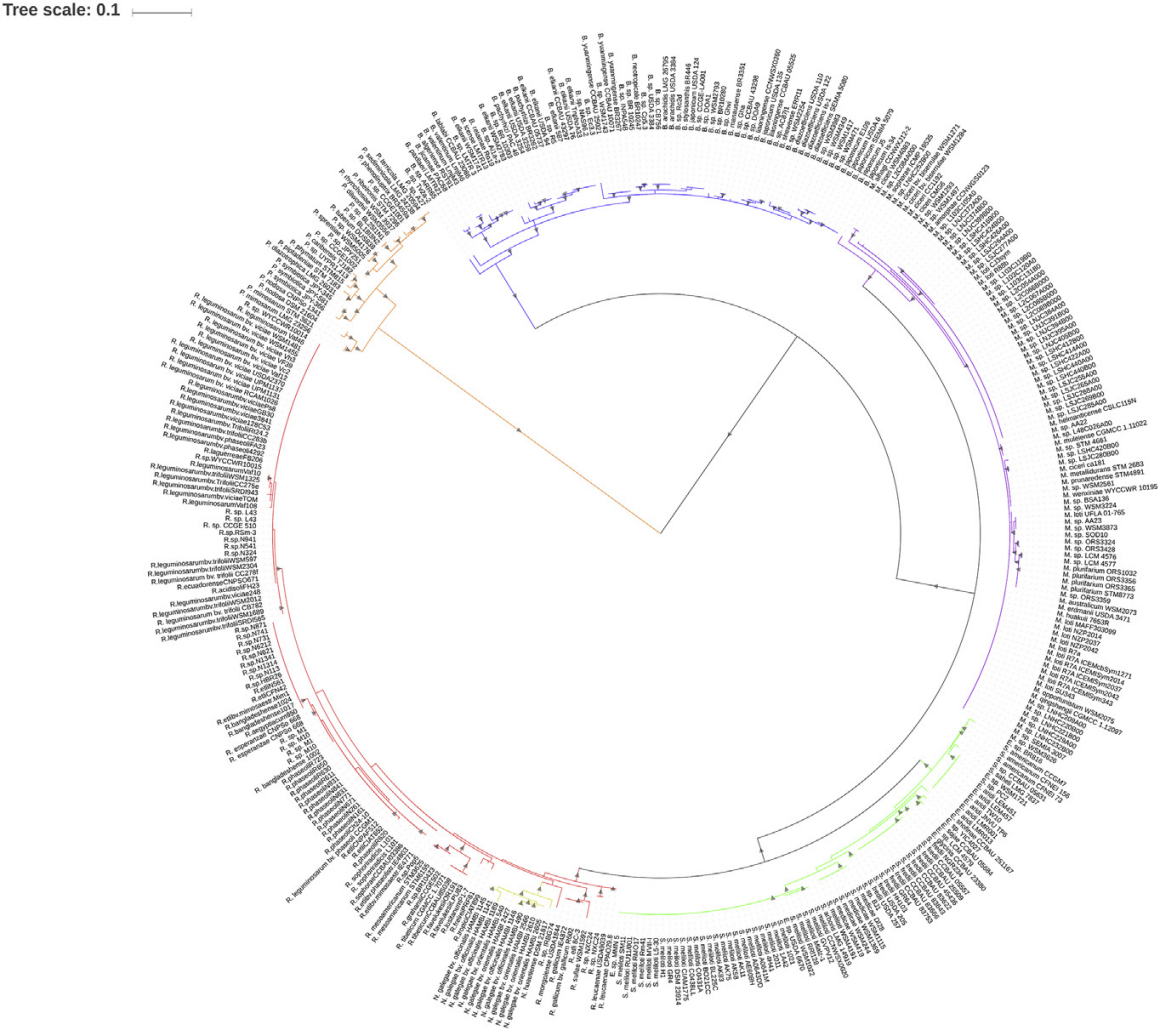
Phylogram based on RecA sequences from rhizobia. The evolutionary history was inferred by using the Maximum Likelihood method based on the Le and Gascuel model (Le and Gascuel, 1993). The tree with the highest log likelihood (-4347.6378) is shown. The percentage of trees in which the associated taxa clustered together is shown next to the branches. Initial tree(s) for the heuristic search were obtained by applying the Neighbor-Joining method to a matrix of pairwise distances estimated using a JTT model. A discrete Gamma distribution was used to model evolutionary rate differences among sites (5 categories (+G, parameter = 0.4656)). The rate variation model allowed for some sites to be evolutionarily invariable ([+I], 34.1847% sites). The tree is drawn to scale, with branch lengths measured in the number of substitutions per site. The presence of grey triangles in the branches indicates a bootstrap support from 50 to 100%. *Bradyrhizobium*- blue branches; *Paraburkholderia*- orange branches; *Mesorhizobium*- purple branches; *Rhizobium*- red branches; *Sinorhizobium/Ensifer*- green branches; *Neorhizobium*- light brown branches.

The phylogram based on the 395 identified NodC sequences (Fig. 1) shows a grouping that is mostly independent of the rhizobial species (incongruent to the obtained RecA-based phylogeny, Fig. 2). This suggests that NodC is subjected to increased mutational rates and/or has mainly evolved through horizontal gene transfer (HGT) events. This is consistent with the observed increased evolutionary distances of NodC compared to those of RecA in the distinct rhizobial groups (Table 1).

**Table 1:** Prevalence, localization and evolutionary distances of NodC, AcdS and RtxC in distinct rhizobial genera.

Genera	*nodC* location in the replicon	AcdS+	*acdS* location in the replicon	RtxC+	*rtxC* location in the replicon	Evolutionary Distances							
						RecA All	RecA (AcdS+)	RecA (RtxC+)	NodC All	NodC (AcdS+)	NodC (RtxC+)	AcdS	RtxC
*Bradyrhizobium*	Chromosome (symbiotic island)	71/71	Chromosome	33/71	Chromosome (symbiotic island)	0.032 ±0.005	0.032 ±0.005	0.026 ±0.005	0.228 ±0.018	0.228 ±0.018	0.130 ±0.012	0.083 ±0.010	0.088 ±0.010
*Paraburkholderia*	Plasmid	27/27	2^nd^ chromosome; plasmid (2^nd^ gene)	8/27	Plasmid	0.040 ±0.007	0.040 ±0.007	0.029 ±0.007	0.290 ±0.022	0.290 ±0.022	0.101 ±0.011	0.093 ±0.011	0.086 ±0.010
*Mesorhizobium*	Chromosome (symbiotic island), Plasmid	47/96	Chromosome (Symbiotic island), plasmid	0	-	0.010 ±0.003	0.011 ±0.003	-	0.231± 0.018	0.256 ±0.021	-	0.134 ±0.012	-
*Rhizobium*	Plasmid	37/104	Plasmid	3/104	Plasmid	0.026 ±0.005	0.032 ±0.006	0.005 ±0.003	0.264 ±0.022	0.287 ±0.021	0.019 ±0.005	0.284 ±0.025	0 ±0
*Sinorhizobium/Ensifer*	Plasmid	39/71	Plasmid	6/71	Plasmid	0.023 ±0.005	0.022 ±0.004	0.029 ±0.006	0.211 ±0.017	0.203 ±0.016	0.172 ±0.018	0.215 ±0.022	0.151 ±0.018
*Neorhizobium*	Plasmid	0/11	**-**	0/11	-	-	-	-	-	-	-	-	-
*Azorhizobium*	Chromosome (symbiotic island)	2/2	Chromosome	0/2	-	-	-	-	-	-	-	-	-
*Allorhizobium*	Plasmid	1/1	**-**	0	-	-	-	-	-	-	-	-	-
*Methylobacterium*	Chromosome	3/3	Chromosome	0	-	-	-	-	-	-	-	-	-
*Microvirga*	unknown	3/3	unknown	0	-	-	-	-	-	-	-	-	-
*Cupriavidus*	Plasmid	4/5	unknown	0	-	-	-	-	-	-	-	-	-

- not studied.

### 2.2 Prevalence and evolutionary analysis of AcdS and RtxC in NodC-containing genomes

#### 2.2.1 ACC deaminase

ACC deaminase sequences were found in NodC-containing Alpha and Betaproteobacteria (Table 1, Fig. 1). Of 395 rhizobial genomes, 234 (59.2%) possessed ACC deaminase. However, the prevalence of AcdS greatly varied between different bacterial groups (Table 1). The AcdS was identified in 71 out of 71 (100%) *Bradyrhizobium*; 27 of 27 (100%) *Paraburkholderia* strains; 39 of 71 (54.9%) *Sinorhizobium/Ensifer* strains; 47 of 96 (48.9%) *Mesorhizobium* strains; in 37 of 104 (35.6%) *Rhizobium* strains; 4 of 5 *Cupriavidus* strains; 3 of 3 *Methylobacterium* strains; 3 of 3 *Microvirga* strains; 2 of 2 *Azorhizobium* strains; and 1 of 1 *Allorhizobium* strains. The *acdS* gene was not found in *Neorhizobium* and *Pararhizobium* strains*.*

**Fig. 3:**
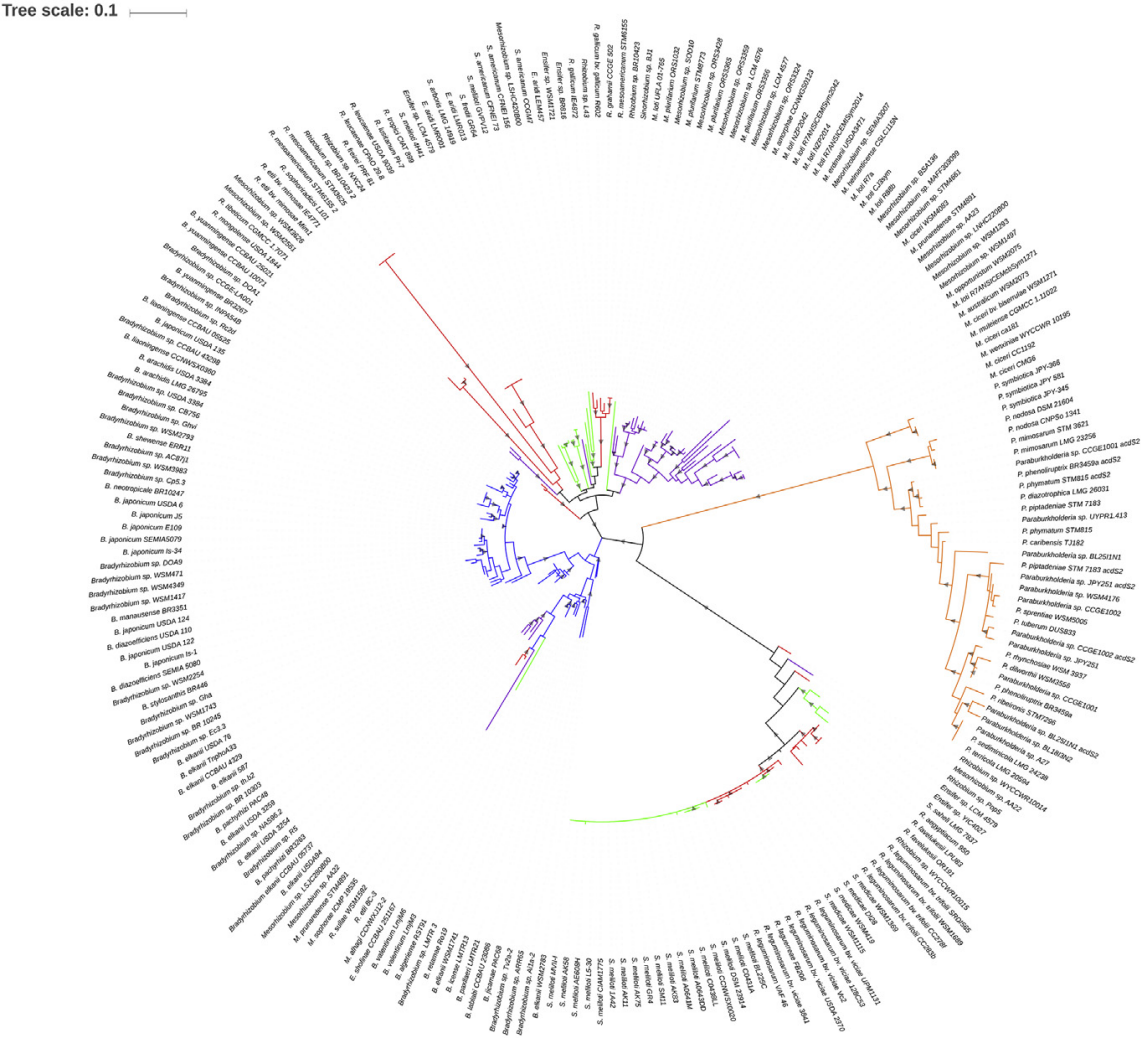
Phylogram based on AcdS sequences from rhizobia. The evolutionary history was inferred by using the Maximum Likelihood method based on the Le and Gascuel model (Le and Gascuel, 1993). The tree with the highest log likelihood (-9509.97) is shown. Initial tree(s) for the heuristic search were obtained by applying the Neighbor-Joining method to a matrix of pairwise distances estimated using a JTT model. A discrete Gamma distribution was used to model evolutionary rate differences among sites (5 categories (+G, parameter = 0.4731)). The tree is drawn to scale, with branch lengths measured in the number of substitutions per site. The presence of grey triangles in the branches indicates a bootstrap support from 50 to 100%. *Bradyrhizobium*- blue branches; *Paraburkholderia*- orange branches; *Mesorhizobium*- purple branches; *Rhizobium*- red branches; *Sinorhizobium/Ensifer*- green branches.

The localization of the *acdS* gene in the replicon varied in the different rhizobial groups (Table 1). *Bradyrhizobium* and *Paraburkholderia* strains possess the *acdS* gene in chromosomal regions, away from the symbiotic islands or plasmids containing the symbiosis genes (*nod*, *nif*). In these genera, AcdS is highly prevalent (Fig. 1, Table 1) and the evolutionary distances of AcdS mostly mimic those of the housekeeping RecA (Table 1), suggesting that, in these bacteria, ACC deaminase mostly evolved vertically and stably (in the chromosome) throughout time. This is also observed in the AcdS-based phylogram, where *Bradyrhizobium* and *Paraburkholderia* form strong independent clusters (Fig. 3).

On the other hand, *acdS* prevalence is lower in *Rhizobium*, *Sinorhizobium/Ensifer* and *Mesorhizobium* strains, which possess the *acdS* gene in mobile genetic elements including plasmids and symbiotic islands (Fig. 1; Table 1). These rhizobial groups present an increased AcdS evolutionary distance, not mimicking RecA evolution, but mimicking NodC evolution instead (Table 1). This result indicates that in these strains AcdS evolved mainly by HGT events by the integration of the *acdS* gene in plasmids that also contained the symbiotic genes. This is observed in the AcdS-based phylogram (Fig. 3), showing that the AcdS from these genera does not group according to the bacterial genus/species but rather are dispersed throughout the phylogram (which is also observed in the NodC-based phylogeny). Moreover, the AcdS phylogram also shows recent HGT events between *Rhizobium*, *Mesorhizobium* strains and *Bradyrhizobium* (donor) (Fig. 3). Additionally, a significant correlation was found between the presence AcdS and the plant host of the recipient rhizobial strain, hence, suggesting that the plant host plays a role in the selection of *acdS* genes in the rhizobial population. This is clearly observed in Fig. 1.

#### 2.2.2 Dihydrorhizobitoxine desaturase

The RtxC is significantly less abundant than AcdS amongst NodC^+^ rhizobia. Its presence is detected in 33 of 71 (45.1%) *Bradyrhizobium*; 8 of 27 (33.3%) *Paraburkholderia* strains; 6 of 71 (8.4%) *Sinorhizobium/Ensifer;* and 3 of 104 (3%) *Rhizobium* strains. No RtxC is found in *Mesorhizobium* or other rhizobial groups. This result indicates that RtxC is negatively selected in most symbioses.

**Fig. 4:**
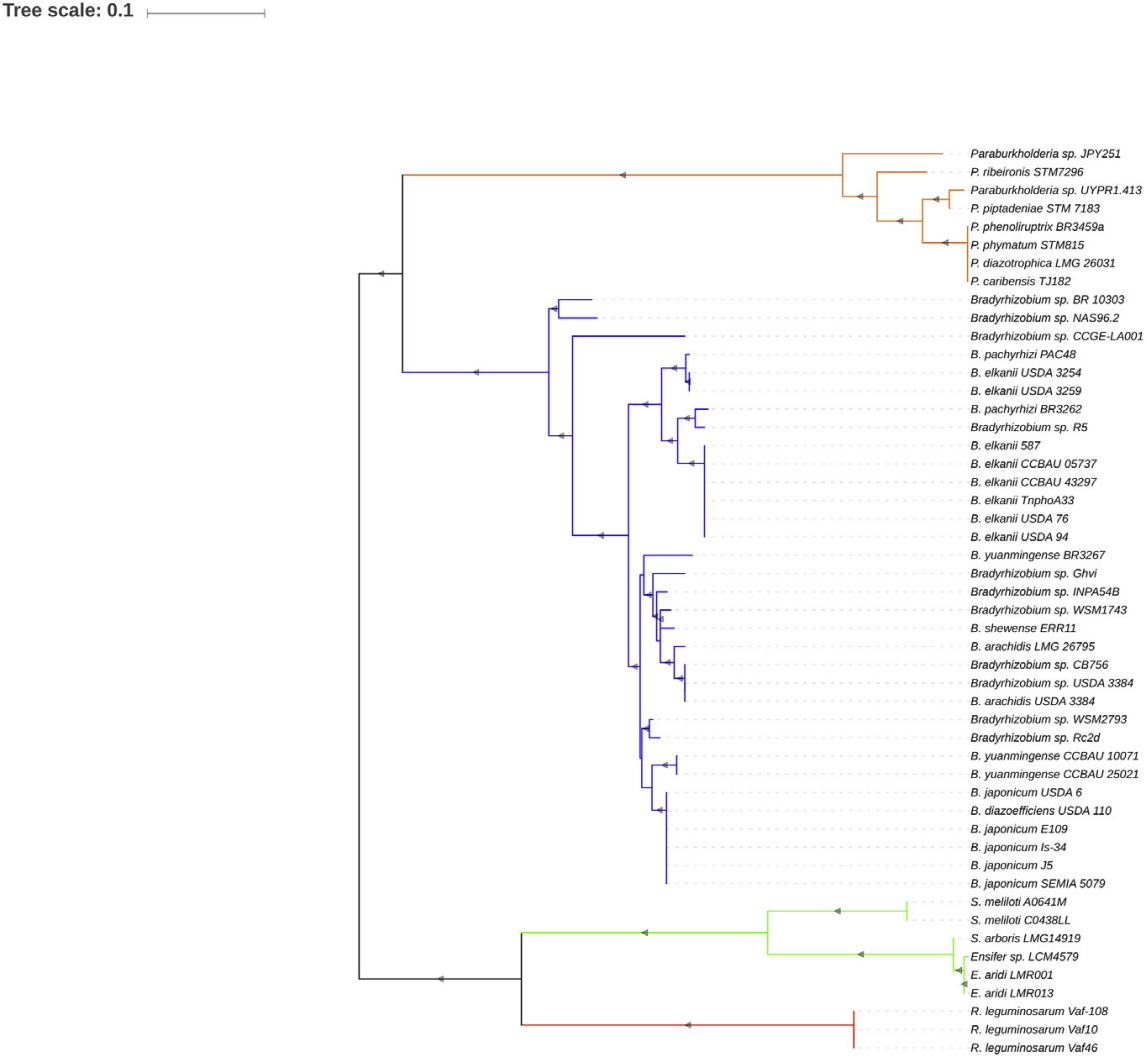
Phylogram based on RtxC sequences from rhizobia. The evolutionary history was inferred by using the Maximum Likelihood method based on the Le and Gascuel model (Le and Gascuel, 1993). The tree with the highest log likelihood (-3988.1676) is shown. Initial tree(s) for the heuristic search were obtained by applying the Neighbor-Joining method to a matrix of pairwise distances estimated using a JTT model. A discrete Gamma distribution was used to model evolutionary rate differences among sites (5 categories (+G, parameter = 1.4551)). The tree is drawn to scale, with branch lengths measured in the number of substitutions per site. The presence of grey triangles in the branches indicates a bootstrap support from 50 to 100%. *Bradyrhizobium*- blue branches; *Paraburkholderia*- orange branches; *Rhizobium*- red branches; *Sinorhizobium/Ensifer*- green branches.

Genome analysis revealed that *rtxC* is typically found in mobile genetic elements in the different rhizobial groups. In *Bradyrhizobium*, the *rtxC* gene is found in the chromosomal symbiotic island near the symbiosis genes. In *Paraburkholderia* and other rhizobia, the *rtxC* gene is found on symbiotic plasmids (Table 1). Still, despite the fact that the *rtxC* is found in mobile genetic elements, evolutionary analysis of all rhizobial groups show that RtxC is more conserved than NodC, suggesting that the RtxC acquisition is recent or, alternatively, that RtxC is not prone to high mutational rates. Curiously, the RtxC-based phylogram (Fig. 4) shows that RtxC from *Bradyrhizobium* and *Paraburkholderia* group close together, away from *Rhizobium* and *Sinorhizobium/Ensifer* RtxC. This suggests past HGT events between *Bradyrhizobium* and *Paraburkholderia,* and an independent acquisition of *rtxC* in other rhizobia.

A significant correlation between the presence of RtxC and the NodC group/host of bacteria was found, indicating that the presence of RtxC in rhizobia is related to the ability to nodulate a specific host plant. For example, RtxC is most often found in *Bradyrhizobium* strains nodulating soybean (*Glycine max*), *Paraburkholderia* strains nodulating *Mimosa*, *Rhizobium* strains nodulating *Vavilovia formosa*, and *Sinorhizobium*/*Ensifer* strains nodulating *Prosopis*, *Acacia* and *Medicago sativa cv. Oneida* (Fig. 1). Interestingly, most strains possessing RtxC also possess AcdS. This result suggests that the nodulation process of these strains is highly regulated by ethylene, thus, providing an advantage to rhizobial strains that possess both of these ethylene-limiting mechanisms.

## 3 Discussion

### 3.1 Ethylene modulation mechanisms, especially ACC deaminase, are relevant for the evolution of nodulation abilities in rhizobia

*Bradyrhizobium* spp. and *Paraburkholderia* spp. are ancient, and, very close to the precursors of the nodulation process in Proteobacteria (Aoki et al., 2013; Bontemps et al., 2010; Martinez-Romero, 2009; Ormeño-Orrillo et al., 2013). The divergence between Bradyrhizobia and fast-growing rhizobia occurred long ago, and the divergence between Alpha and Betaproteobacteria is even older (Chriki-Adeeb and Chriki, 2016; Turner and Young, 2000). It is therefore likely that older *Bradyrhizobium* spp. and *Paraburkholderia* spp. closely interacted with plants for a long time before the appearance of nodulation genes. This is consistent with the fact that nitrogen fixation is common in these genera, and that *nifH* genes (which are older than *nod* genes) from *Paraburkholderia* strains show higher homology to *nifH* genes from *Bradyrhizobium* than to other alphaproteobacterial rhizobial strains (Bontemps et al., 2010).

Interestingly, several results obtained in this study support the idea that *Bradyrhizobium* spp. and *Paraburkholderia* spp. are ancient symbionts: A) compared to other rhizobia, *Bradyrhizobium* spp. and *Paraburkholderia* spp. NodC sequences cluster closer together (even if these bacteria were obtained from different locations and plants), and NodC evolution in these genera is more stable and less prone to distant HGT events; B) ACC deaminase genes are highly prevalent and are stably vertically transmitted in *Bradyrhizobium* spp. and *Paraburkholderia* spp. (including non-symbionts, Nascimento et al., 2014) but not in other rhizobia; C) The prevalence of RtxC is increased in these groups but not in other rhizobia, and RtxC from *Bradyrhizobium* strains show higher homology to RtxC from *Paraburkholderia* than to RtxC from other alphaproteobacteria rhizobia strains.

The presence of AcdS in *Bradyrhizobium* spp. (including ancient photosynthetic Bradyrhizobia) and *Paraburkholderia* spp. (symbionts, PGPB and closely related *Burkholderia* pathogens) appears to result from a previous ancestral selection relating to the ability to internally colonize plants and modulate plant ethylene levels responsible for regulating the basal plant defense response (Nascimento et al., 2018), that dates to a time before the appearance of nodulation abilities in these genera (pre-symbiotic stage) and possibly even before leguminous plant evolution. This is consistent with the fact that *Bradyrhizobium* spp. and *Paraburkholderia* spp. are commonly found in the endophytic compartment of several non-leguminous plant species (Chaintreuil et al., 2000; Onofre-Lemus et al., 2009), and that ACC deaminase is an ancient mechanism found in older plant-associated microorganisms such as Actinobacteria and Fungi (Nascimento et al., 2014). The fact that both *Bradyrhizobium* spp. and *Paraburkholderia* spp. possessed ACC deaminase may have facilitated the later development of a successful symbiotic relationship, by the modulation of ethylene and ACC levels involved in regulating the plant defense and abiotic stress response (which evolved before the nodulation process), as well as, the symbiotic program. This possibility is consistent with several evidences: rhizobial ACC deaminase minus mutants form fewer nodules and are less competitive than their wild-type counterparts (Ma et al., 2003; Uchiumi et al., 2004); *P. phymatum* STM815 expresses the ACC deaminase gene soon after its contact with *M. pudica* root exudates (Klonowska et al., 2018), some symbiotic *Paraburkholderia* strains possess two *acdS* genes, one of these is present in the symbiotic plasmid and is a result of a gene duplication event (Nascimento et al., 2014), thus indicating a strong selective pressure to maintain this gene.

RtxC is more prevalent in *Bradyrhizobium* and *Paraburkholderia* than in other rhizobia, and the presence of RtxC in these groups seems to be related to the ability to nodulate specific hosts. Moreover, the results obtained in this work show that RtxC are mostly found in symbiotic islands and symbiotic plasmids, suggesting an initial acquisition by HGT, integration in mobile elements and a subsequent evolution. Rhizobitoxine is structurally very similar to aminoethoxyvinylglycine (AVG), a metabolite produced by *Streptomyces,* a member of the ancient Actinobacteria phylum, and possibly the donor of the precursor genes. Curiously, *rtxC* genes are mostly found in plant pathogens such as *Xanthomonas* spp. (Alphaproteobacteria), *Burkholderia spp.* (Betaproteobacteria) and *Pseudomonas syringae* (Gammaproteobacteria) (Nascimento et al., 2018). It is possible that RtxC was acquired by these bacteria before the appearance of the nodulation process. Some symbionts seem to have acquired and maintained *rtxC* has a result of a positive selection pressure possibly induced by the host plant (discussed below). So, ultimately, rhizobitoxine production may have impacted the development of the nodulation process, especially in *Bradyrhizobium* and *Paraburkholderia*, although its role seems to be more limited and specific than AcdS.

On the other hand, more recently evolved rhizobia (e.g. *Rhizobium*, *Sinorhizobium*, *Mesorhizobium*) seem to have acquired ACC deaminase genes, in some cases by HGT by plasmid and symbiotic island transfer (Hontzeas et al., 2005; Nascimento et al., 2012). In these strains, the *acdS* gene is present and maintained near the *nod* and *nif* genes in the symbiotic islands and/or plasmids. In some *Rhizobium* and *Sinorhizobium*/*Ensifer* strains, two *acdS* genes (duplication or HGT) can also be found, thus, indicating a strong positive selective pressure for this gene. In several rhizobial strains, *acdS* gene expression is regulated by NifA (a transcriptional regulator of nitrogen fixation genes) (Nascimento et al., 2012; Nukui et al., 2006), which provides evidence for the close evolutionary relationship between ACC deaminase expression and nitrogen fixation. Hence, acquiring and maintaining ACC deaminase genes benefited the symbiotic process induced by these strains (Ma et al., 2003; Uchiumi et al., 2004). Contrarily, it seems that *rtxC* was mostly lost and negatively selected in these symbionts.

### 3.2 Co-evolution with a plant host and consequent HGT events as the main factors involved in the selection and evolution of ET modulation genes

Significant correlations between the presence of AcdS and RtxC and the NodC group/host of the bacteria indicates that, in some cases, the presence ethylene modulation genes is related to the ability to nodulate a specific host plant. Hence, it seems that some plants induce the selection of ethylene modulation genes in their rhizobial symbionts.

Some leguminous plants possess a relatively large number of genetic elements involved in ethylene production and sensitivity compared to other plants (Desbrosses and Stougaard, 2011), and this is reflected in their overall production and response to ACC and ethylene (Miyata et al., 2013; Penmetsa et al., 2008). For example, *Glycine max* possesses fourteen ACC synthase genes while most other leguminous plants possess only six ACC synthase genes. Similarly, most leguminous plants contain one or two copies of the EIN2 gene (involved in ethylene signaling) while *G. max* possesses six EIN2 gene copies. Curiously, *G. max* presents a decreased sensitivity to exogenous ACC and ethylene (Schmidt et al., 1999), which may be related to its ability to produce more ACC and ethylene when compared to other leguminous plants. Not surprisingly, most *G. max Bradyrhizobia* possess both RtxC and AcdS.

Environmental stress conditions induce ACC and ethylene production in plants. Hence, leguminous plant hosts subjected to stress may produce increased ACC and ethylene levels and induce the selection of ACC-degrading and RtxC-producing rhizobia. The stress-induced selection of ACC deaminase-producing bacteria has been described in other plants (reviewed by Nascimento et al., 2018).

Additionally, the presence of a specific plant host can induce the indirect selection of AcdS and RtxC, by inducing the selection of specific plasmids and symbiotic islands containing a specific set of compatible symbiotic genes. For example, it has been shown that *Mesorhizobium opportunistum* WSM 2073 gained the ability to nodulate *Biserrula pelecinus* by acquiring a specific symbiotic island when it came in contact with introduced non-endemic populations of *M. ciceri* bv. *biserrulae* (Nandasena et al., 2007). The *acdS* gene was present within that symbiotic island and, consequently, was transferred between the strains. This result also indicates that the presence of compatible *acdS* and *rtxC* donors in the soil/rhizosphere bacterial community plays a role in the subsequent transfer and evolution of these genes.

## 4 Conclusions

Ethylene modulation genes, *acdS* and *rtxC*, are widely present in NodC-containing rhizobia belonging to distinct genera, which is consistent with the important role of ethylene in regulating the symbiotic process of most leguminous plants. These mechanisms have evolved differently in various rhizobial populations, and this appears to be dependent on several factors such as the unique evolution of each rhizobial group, the pool of *acdS* and *rtxC* in the rhizobial community, and HGT events. Importantly, the plant host plays a role in the selection of ethylene-modulating genes in the rhizobial population, possibly by its direct effect on the symbiont through ACC and ethylene production and sensitivity (activation of defense and symbiotic programs; stress conditions induced by the environment), or indirectly, by the induced selection (by specific flavonoids) of plasmids containing specific sets of *nod* genes and the nearby *acdS* and/or *rtxC* that comes associated.

## 5 Materials and methods

### 5.1 Obtaining the rhizobial sequences

The protein NodC encodes an N-acetylglucosaminyltransferase enzyme that is responsible for the production of specific NFs involved in the elicitation of the legume symbiotic response. Hence, the NodC was selected as a marker for rhizobia nodulation abilities, and its adaptation to specific plant hosts.

A BlastP analysis (default parameters) was performed to study the presence of NodC in bacterial genomes deposited in the NCBI database (www.ncbi.nlm.nih.gov). The NodC sequences from *Bradyrhizobium diazoefficiens* USDA 110 (WP_011084824.1), *Mesorhizobium* sp. MAFF303099 (WP_010913821.1), *Rhizobium leguminosarum* bv. *viciae* 3841 (WP_003540131.1), and *Paraburkholderia phymatum* STM815 (WP_012406749.1) were used as queries in the BlastP analysis.

Subsequently, bacteria possessing NodC were then selected for future studies based on the prevalence of ACC deaminase (AcdS) and/or dihydrorhizobitoxine desaturase (RtxC). *Bradyrhizobium diazoefficiens* USDA 110 functional AcdS (WP_011083073.1) and RtxC (WP_011084875.1) sequences were used as queries in BlastP searches (default parameters) in the NCBI database.

The DNA recombinase A (RecA) protein sequence (chosen as a representative protein sequence of a housekeeping and vertically transmitted gene) from the NodC-containing rhizobia, as well as strain information (host isolation, geographical location) were obtained from the NCBI database. The RecA, NodC, AcdS and RtxC protein sequences were further used in phylogenetic and evolutionary distances analysis (described below).

The gene location analysis (chromosome vs. plasmids) was performed in selected completely sequenced rhizobial genomes present in the NCBI database.

All sequence accession numbers and strain information is presented in Table S1.

### 5.2 Phylogenetic analysis and evolutionary distance calculations

The protein sequences were aligned using MUSCLE (Edgar, 2004) and then used in phylogenetic and evolutionary distance analyses.

Phylogenetic analysis and evolutionary distance calculations were performed using MEGA v.6.0.6 (Tamura et al., 2013). The best Maximum Likelihood (ML) model for each protein alignment was selected based on the lower Bayesian Information Criterion values obtained from an analysis with the Mega ML model selection tool, using default parameters.

The phylogenetic analysis was performed using the ML method, the appropriate selected model (described in the figure caption of each phylogram), and a bootstrap method analysis with 250 replications.

The obtained newick files were uploaded to the Interactive Tree of Life (iTOL) v.3 website (http://itol.embl.de) (Letunic and Bork, 2016) and phylograms were generated, edited and analyzed.

Evolutionary distance calculations were performed in MEGA v.6.0.6, using the Bootstrap method with 500 replications and the Jones-Taylor-Thornton (JTT) matrix-based substitution model (Jones et al., 1992). The rate of variation among sites was modeled with a Gamma distribution (shape parameter = 5).

Phylogenetic analysis and evolutionary distance calculations were performed using sequences from the rhizobial groups containing more than 10 available genomes.

### 5.3 Statistical correlations

Statistical correlation analyses were performed using IBM SPSS Statistics v.22 with parametric (Pearson correlation parameter, two-tailed) and non-parametric models (Kendal's tau B and Spearman, two tailed). The correlations presented in this study were significant at the 0.01 level (2-tailed) by all of the tested methods.

## Declarations

### Author contribution statement

Francisco X. Nascimento: Conceived and designed the experiments; Performed the experiments; Analyzed and interpreted the data; Wrote the paper.

Maria J. Tavares: Analyzed and interpreted the data; Contributed reagents, materials, analysis tools or data; Wrote the paper.

Márcio J. Rossi, Bernard R. Glick: Contributed reagents, materials, analysis tools or data; Wrote the paper.

### Funding statement

Márcio J. Rossi was supported by Conselho Nacional de Desenvolvimento Científico e Tecnológico (CNPq), Brazil (DT 306167/2015-8).

### Competing interest statement

The authors declare no conflict of interest.

### Additional information

No additional information is available for this paper.

## Appendix A Supplementary data

**Table S1** Sequence accession numbers and strains information.
